# Particularités épidémio-cliniques, biologiques et radiologiques de la tuberculose pulmonaire chez les diabétiques à Antananarivo, Madagascar

**DOI:** 10.11604/pamj.2022.42.49.29199

**Published:** 2022-05-18

**Authors:** Miora Maëva Arielle Andrianiaina, Rija Eric Raherison, Thierry Razanamparany, Sitraka Angelo Raharinavalona, Andrianirina Dave Patrick Rakotomalala, Radonirina Lazasoa Andrianasolo

**Affiliations:** 1 Service d´Endocrinologie du Centre Hospitalier Universitaire Joseph Raseta Befelatanana Antananarivo, Antananarivo, Madagascar,; 2 Service de Médecine du Centre Hospitalier Régional de Référence de Vakinakaratra Antsirabe, Vakinakaratra Antsirabe, Madagascar,; 3 Service de Médecine Interne et des Maladies Cardiovasculaires du Centre Hospitalier de Soavinandriana, Soavinandriana, Madagascar

**Keywords:** Clinique, diabète sucré, Madagascar, prévalence, radiologie, tuberculose pulmonaire, Clinic, diabetes mellitus, Madagascar, prevalence, radiology, pulmonary tuberculosis

## Abstract

La tuberculose pulmonaire est fréquemment associée au diabète sucré, avec parfois des présentations atypiques. Notre objectif était d´identifier les particularités épidémiologiques, cliniques, biologiques et radiologiques de la tuberculose pulmonaire chez les diabétiques par rapport aux non diabétiques. Ceci afin d´améliorer leur prise en charge. l´étude a été réalisée dans les services d´Endocrinologie et de Pneumologie du Centre Hospitalier Universitaire Joseph Raseta Befelatanana et le Service de Maladies Respiratoires du Centre Hospitalier de Soavinandriana à Antananarivo, Madagascar. Elle a concerné les cas de tuberculose pulmonaire à bacilloscopie positive enregistrés de janvier 2018 à janvier 2020 (25 mois). C´est une étude rétrospective transversale descriptive et analytique. Dans notre étude, la prévalence du diabète chez les tuberculeux était de 20,31%. Un âge plus avancé, une évolution clinique à bas bruit, un syndrome inflammatoire biologique important, moins de cavernes mais plus d´opacités systématisées, des lésions radiologiques à localisation basale ou diffuses, plutôt unilatérales droites, ont été constatés plus fréquemment chez les diabétiques comparés aux non diabétiques. La connaissance des particularités de la présentation de la tuberculose pulmonaire chez les diabétiques aidera les personnels de santé à ne pas passer à côté du diagnostic même devant une présentation atypique. Par ailleurs, comme la majorité des tuberculoses pulmonaires survient chez les diabétiques déséquilibrés, un bon équilibre glycémique permettra certainement de diminuer son incidence.

## Abstract (en)

Pulmonary tuberculosis is frequently associated with diabetes mellitus and, sometimes, it may present with atypical signs. The purpose of this study is to identify the epidemiological, clinical, biological and radiological features of pulmonary tuberculosis in patients with diabetes versus patients without diabetes in order to improve treatment. We conducted a retrospective, cross-sectional descriptive and analytical study in the Department of Endocrinology and Pneumology at the Joseph Raseta Befelatanana University Hospital Center and in the Department of Respiratory Diseases at the Soavinandriana Hospital Center in Antananarivo, Madagascar. It involved patients diagnosed with pulmonary tuberculosis who presented a positive bacilloscopy exam from January 2018 to January 2020 (25 months). In our study, the overall prevalence of diabetes among TB patients was 20.31 %. Older age, insidious clinical course, severe biologic inflammatory syndrome, fewer caves but more systematized opacities and diffuse radiological lesions in the basal zone (more commonly unilateral right lesions) were mainly detected in patients with diabetes compared to patients without diabetes. Knowledge of signs of pulmonary TB in patients with diabetes can help health care workers to make a diagnosis, even in patients with atypical signs. Moreover, as the majority of pulmonary tuberculosis occur in patients with unstable diabetes, a good glycemic balance can certainly reduce its incidence.

## Introduction

La tuberculose (TB) pulmonaire correspond à la maladie due à la localisation pulmonaire du *Mycobacterium tuberculosis* ou *Bacille de Koch (BK)*. Elle sévit sur le mode endémique à Madagascar [1]. Le diabète, quant à lui, est caractérisé par une hyperglycémie chronique due à des troubles du métabolisme glucidique [2]. Il devient une maladie pandémique et la population Malgache n´y échappe pas [3]. Il expose à des complications métaboliques et cardiovasculaires liées à l´hyperglycémie chronique mais également à des complications infectieuses favorisées par l´altération du système immunitaire qu´il engendre [4,5]. Parmi ces infections figurent la tuberculose. D´après certaines études, les patients diabétiques présentent des formes de tuberculose pulmonaire plus graves, des localisations atypiques et des anomalies radiologiques souvent étendues [6]. Peu de données sont disponibles concernant les particularités de la tuberculose pulmonaire chez les diabétiques par rapport aux sujets non diabétiques à Madagascar. C´est pourquoi nous avons réalisé cette étude ayant pour objectif de déterminer les particularités épidémio-cliniques, biologiques et radiologiques de la tuberculose pulmonaire chez les sujets diabétiques par rapport aux non diabétiques à Antananarivo, Madagascar. Ceci afin d´optimiser son dépistage et sa prise en charge.

## Méthodes

Il s´agissait d´une étude rétrospective, descriptive et analytique, multicentrique réalisée au sein des services d´Endocrinologie et de Pneumologie du Centre Hospitalier Universitaire Joseph Raseta Befelatanana, (CHUJRB) et du service des Maladies Respiratoires du Centre Hospitalier de Soavinandriana, (CENHOSOA) entre le 1^er^ janvier 2018 et le 31 janvier 2020 (25 mois). Pour être inclus dans l´étude, les patients devraient être âgés de plus de 15 ans et hospitalisés pour tuberculose pulmonaire à bacilloscopie positive (TPB+) confirmée soit par la présence de Bacillles Acido-Alcoolo résistants (BAAR) dans les crachats soit par la positivité du test geneXpert des crachats et ayant réalisé au moins deux dosages de glycémie à jeun et/ou un dosage de l´hémoglobine glyquée (HbA1c) permettant de distinguer les patients diabétiques des patients non diabétiques.

Ont été exclus les dossiers incomplets rendant les résultats de l´analyse des données inexploitables. Les données anamnestiques, cliniques, biologiques et radiologiques ont été recueillies et comparées chez les TPB+ diabétiques et les TPB+ non diabétiques. L´analyse statistique a consisté en une description et une comparaison de la répartition des patients selon chaque variable étudiée chez les 2 groupes afin d´en tirer les particularités chez les TPB+ diabétiques. Les données ont été analysées par le logiciel Epi-Info version 7.2.2.6 et les tests statistiques sont retenus significatifs pour une valeur de p < 0,05.

## Résultats

Parmi les 410 patients TPB+ inclus dans les 3 sites, trois cent vingt (78,05%) ont été retenus pour l´étude comparative finale, se répartissant en 65 diabétiques (20,31%) (n1) et 255 non diabétiques (79,69%) (n2). Les diabétiques étaient plus âgés par rapport aux non diabétiques avec des âges moyens respectifs de 53,05 ± 12,89 ans (18 à 79 ans) et 38,56 ± 15,19 ans (16 à 85 ans) (p < 0,01). Le sex ratio était de 1,5 pour les diabétiques et de 2,27 pour les non diabétiques (p = 0,14). Un antécédent de TB a été retrouvé chez 26,42% des diabétiques et 16,08% des non diabétiques (p = 0,5). Une notion de contage tuberculeux était signalée par 18,46% des diabétiques et 29,02% des non diabétiques (p = 0,08). La durée moyenne d´évolution des symptômes avant l´hospitalisation était de 39,81 ± 59,25 jours chez les diabétiques et de 86,02 ± 108,92 jours chez les non diabétiques (p <0,01). Cliniquement, l´Indice de Masse Corporelle (IMC) moyen des diabétiques était de 18,54 ± 3,78 kg/m^2^ (11 à 27 kg/m^2^) et celui des non diabétiques de 16,29 ± 2,01kg/m^2^ (10,70 à 23,18 kg/m^2^) et la différence était statistiquement significative (p <0,01). Sur le plan clinique, les principaux signes présentés par les patients se résumaient dans le Tableau 1.

**Tableau 1:** répartition des patients selon les signes cliniques (n1 = 65, n2 = 255)

Signes	TPB+ diabétiques n1	TPB+ non diabétiques n2	p-value
**Signes fonctionnels**			
Toux grasse	40(61,54%)	209(81,96%)	< 0,01
Toux sèche	16(24,62%)	99(38,82%)	0,03
Dyspnée	16(24,62%)	117(45,88%)	<0,01
Hémoptysies	8(12,31%)	75(29,41%)	< 0,01
Douleur thoracique	15(23,08%)	57(22,35%)	0,9
Néant	11(16,92%)	4(2,04%)	<0,01
**Signes généraux**			
Asthénie	48(73,85%)	200(78,43%)	0,43
Amaigrissement	40(61,54%)	194(76,08%)	0,02
Anorexie	34(52,31%)	186(72,94%)	0,01
Fièvre	36(55,39%)	167(65,49%)	0,13
Sudations nocturnes	14(21,54%)	68(26,67%)	0,40
Néant	12(7,69%)	5(1,96%)	0,34
**Signes auscultatoires pulmonaires**			
Râles crépitants	36(55,38%)	178(69,80%)	< 0,01
Râles bronchiques	6 (9,23%)	29(11,37%)	0,62
Souffle caverneux	5(7,69%)	19(7,45%)	0,95
Néant	15(23,08%)	24(9,41%)	< 0,01

TPB+: Tuberculose Pulmonaire à Bacilloscopie positive

**Figure 1:**
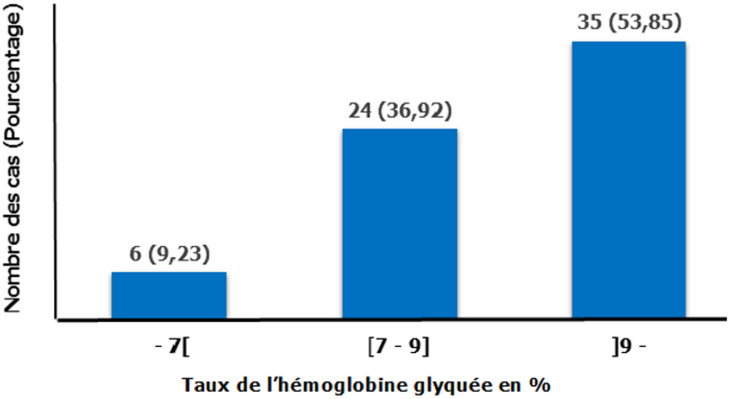
répartition des patients TPB+ diabétiques selon le taux de l´hémoglobine glyquée (n1 = 65); (TPB+: Tuberculose Pulmonaire à Bacilloscopie positive)

Ainsi, parmi les signes fonctionnels respiratoires, la toux grasse, la toux sèche, la dyspnée et les hémoptysies étaient statistiquement moins fréquents chez les diabétiques en comparaison aux non diabétiques et 16,92% des diabétiques étaient asymptomatiques contre 2,04% des non diabétiques. Parmi les signes généraux, l´asthénie, l´amaigrissement et l´anorexie étaient les plus rapportés chez les 2 groupes mais l´amaigrissement et l´anorexie étaient significativement moins fréquents chez les diabétiques. Et parmi les signes auscultatoires pulmonaires, les râles crépitants étaient les signes auscultatoires les plus rapportés chez les 2 groupes. Toutefois, ils étaient moins fréquents chez les diabétiques (55,38%) que les non diabétiques (69,80%) (p < 0,01). Par ailleurs, l´auscultation pulmonaire était normale chez 23,08% des diabétiques et 9,41% des non diabétiques (p < 0,01). Sur le plan biologique, la glycémie à jeun des diabétiques était en moyenne de 243 ± 100 mg/dl (40 à 500 mg/dl) à leur admission. Une glycémie supérieure à 200 mg/dl était retrouvée chez 58,46% d´entre eux. Leur diabète était en général mal équilibré avec un taux d´hémoglobine glyquée (HbA1c) moyen de 8,94 ± 1,81 % (4,6 à 14%). La Figure 1 représentait la répartition des patients diabétiques selon leur taux d´HbA1C.

Par ailleurs, le syndrome inflammatoire reflété par l´élévation de la valeur de C-Reactive Protein (CRP) était plus important chez les diabétiques par rapport aux non diabétiques. De même, la fonction rénale estimée par le Débit de Filtration Glomérulaire estimée (DFGe) à partir de la formule du Chronic Kidney Disease - Epidemiology Collaboration (CKD-EPI) était moins bonne chez les diabétiques que les non diabétiques (Tableau 2). A la radiographie thoracique, les lésions élémentaires rencontrées par ordre de fréquence décroissante étaient les infiltrats pulmonaires, les nodules, les cavernes, les opacités systématisées et enfin les tuberculomes chez les 2 groupes. Cependant, les opacités systématisées étaient significativement plus fréquentes chez les diabétiques. Notons qu´un patient pouvait présenter plusieurs types de lésions en même temps. Les topographies et les étendues de ces lésions radiologiques se répartissaient comme suit avec prédominance des lésions exclusivement basales, des lésions étendues et des lésions unilatérales droites et une moindre fréquence des lésions apicales et des lésions bilatérales chez les diabétiques. Le Tableau 3 résumait les caractéristiques radiologiques des patients.

**Tableau 2:** répartition des patients selon les caractéristiques biologiques (n1= 65, n2= 255)

Caractéristiques	TPB+ diabétiques n1	TPB+ non diabétiques n2	p-value
Taux de leucocytes (G/l)	10,33±5,8(2,9-43,2)	9,8±4,5(1,4-22,9)	0,61
Taux d´hémoglobine(g/l)	118,26±24,73(50-163)	114,31±26,67(41–196)	0,19
CRP(mg/l)	100,93±79,83(5-373)	76,51±61,29(5–337,1)	0,03
DFG estimé(ml/min/1,73m^2^)	87,46±29,65(32–148)	105,34±29,60(20 – 160)	< 0,01

TPB+: Tuberculose Pulmonaire à Bacilloscopie positive ; CRP: C- réactive protéine; DFG : débit de filtration glomérulaire (estimé selon la formule de CKD-EPI); mg/l: milligramme par litre; ml: millilitre par minute par 1,73 mètre carré de surface corporelle; G/l: giga par litre; g/l: gramme par litre

**Tableau 3:** répartition des patients selon les caractéristiques radiologiques (n1= 65, n2= 255)

Caractéristiques	TPB+ diabétiques n1	TPB+ non diabétiques n2	p-value
**Types de lésions**			
Infiltrats	45(69,23%)	181(70,98%)	0,81
Nodules	33(50,77%)	130(50,98%)	0,98
Cavernes	27(41,54%)	129(50,59%)	0,19
Opacités systématisées	17(26,15%)	18(7,06%)	< 0,01
Tuberculomes	1(1,54%)	6(2,35%)	0,69
**Topographies et étendues des lésions**			
Exclusivement apicales	23(35,38%)	212(83,14%)	< 0,01
Exclusivement basales	17(26,15%)	19(7,45%)	< 0,01
Etendues	15(23,08%)	24(9,41%)	< 0,01
Bilatérales	25(38,46%)	144(56,47%)	< 0,01
Unilatérales droites	29(44,62%)	76(29,8%)	0,02
Unilatérales gauches	11(16,92%)	36(14,12%)	0,56

TPB+: Tuberculose Pulmonaire à Bacilloscopie positive

## Discussion

Dans notre étude, la prévalence du diabète parmi les TPB+ était de 20,31%. Ce qui se rapprochait de celle rapportée par l´équipe de Lawson en Niger en 2017 (23%) [7]. Cette ressemblance serait à liée aux similitudes entre les deux pays. En effet, Madagascar et Niger figurent parmi les pays à forte endémicité tuberculeuse avec une prévalence respective de 413 [8] et 524 cas [7] par 100000 habitants par an. De même, la prévalence du diabète est de 3,9% à Madagascar [9] et de 4,1% à Niger [9] selon les rapports de l´Organisation Mondiale de la Santé (OMS). L´âge plus élevé des diabétiques tuberculeux par rapport aux non diabétiques était également rapporté par d´autres études notamment à Qatar [10] et aux Etats-Unis [11]. En effet, il est connu que le risque de survenue de diabète augmente avec l´âge [12], expliquant en partie ce résultat. Quoi qu´il en soit, ces patients appartenaient encore pour la plupart à la population active et le double fardeau diabète et tuberculose aura des conséquences financières et économiques certaines du faites des coûts directs (frais d´hospitalisation, des examens complémentaires...) et indirects (arrêt de travail, frais de déplacement de la famille...) que doivent supporter la famille, la communauté et le pays. Cette situation est d´autant plus ressentie vu le niveau socio-économique de la population Malgache.

La prédominance masculine des tuberculeux est universelle [12,13]. En effet, les hommes ont plus tendance à fumer du tabac et à boire de l´alcool, les prédisposant à la tuberculose, entre autres [14]. La présence plus fréquente d´antécédent de TB chez les diabétiques pourrait s´expliquer par leur immunité précaire qui les exposerait plus souvent à un nouvel épisode que ce soit par réinfestation ou par réactivation d´une infection tuberculeuse endogène quiescente [15]. La recherche d´un tel antécédent doit être systématique à chaque fois qu´une suspicion de tuberculose se fait sentir chez un diabétique. D´autant plus qu´un antécédent de traitement de tuberculose est reconnu comme l´un des principaux facteurs de résistance aux antituberculeux [16,17]. En effet, les germes peuvent subir une mutation qui les rend résistants aux médicaments [18]. Ceci est d´autant plus dangereux chez les diabétiques puisque ces derniers sont plus à risque de développer une tuberculose multirésistante (TBMR) que les sujets non diabétiques selon une étude réalisée à Shangai [19]. Le délai d´évolution des symptômes plus court chez les diabétiques pourrait s´expliquer par la progression plus rapide de la tuberculose chez eux, avec des signes plus graves et multiples pour ceux qui sont symptomatiques [13]. Ceci serait lié à une réponse inflammatoire accrue au niveau systémique aux antigènes mycobactériens [sécrétion élevée d´interféron gamma (IFN-γ), Interleukine-7 (IL-7), Interleukine-2 (IL-2)…) chez les diabétiques] [20]. La tuberculose, pour ces sujets symptomatiques, aurait alors été découverte plus tôt. De même, la recherche d´un facteur de déséquilibre du diabète conduit souvent à l´hospitalisation et à diverses investigations cliniques et paracliniques à visée étiologique permettant des fois de découvrir l´infection tuberculeuse assez tôt. En effet, vu le niveau d´endémicité de la tuberculose à Madagascar, elle est l´une des causes infectieuses du déséquilibre du diabète que les médecins évoquent beaucoup fréquemment.

Cliniquement, par rapport aux non diabétiques, les signes typiques de tuberculose pulmonaire, à savoir la toux, la dyspnée et les hémoptysies étaient moins fréquents chez les diabétiques. L´équipe de Faurhoult-Jepsen en Tanzanie avait également rapportée cette même tendance en 2012 [21]. De même, la fréquence plus élevée des asymptomatiques sur le plan respiratoire chez les diabétiques par rapport aux non diabétiques était également retrouvée à Taiwan [22]. Deux hypothèses peuvent être avancées pour expliquer cette moindre fréquence des signes respiratoires chez les diabétiques. Primo, le diabète expose à une neuropathie autonome laquelle se développe d´abord au niveau des fibres nerveuses les plus longues tout en sachant que le nerf vague est le nerf autonome le plus long de l´organisme. Or physiologiquement, c´est ce nerf qui innerve les larynx et les voies aériennes basses et donc qui intervient dans le phénomène de toux [23]. Ce qui pourrait altérer le réflexe de toux et, par conséquent, diminuer la fréquence de l´hémoptysie. Secundo, contrairement au niveau systémique, il y aurait une diminution des réponses immunitaires au niveau pulmonaire chez les diabétiques tuberculeux. En effet, certains auteurs avaient rapporté que le liquide de lavage broncho-alvéolaire de diabétiques tuberculeux serait pauvre en macrophages activées [24], ou riche en cytokines anti-inflammatoires tels l´IL-10 mais pauvre en cytokines pro-inflammatoires tels l´IFN-γ [25]. Ce qui pourrait diminuer la fréquence des manifestations purement respiratoires de la tuberculose chez les diabétiques. Quoi qu´il en soit, l´absence de signes fonctionnels pourrait même constituer un danger potentiel parce qu´elle pourrait faire masquer la tuberculose pulmonaire, donc retarder le diagnostic et la prise en charge thérapeutique. L´amaigrissement et l´anorexie étaient moins fréquents chez les diabétiques par rapport aux non diabétiques. Seulement, nous n´avons pas eu connaissance du poids antérieur de la plupart des patients rendant la notion d´amaigrissement subjective. Il se pourrait aussi que les diabétiques soient plus corpulents que les non diabétiques avant même la survenue de la tuberculose. L´anorexie quant à elle pourrait être remplacée par une polyphagie devant un déséquilibre du diabète occasionné ici par l´infection tuberculeuse.

Bien que les râles crépitants soient les signes auscultatoires les plus rapportés chez les 2 groupes, ils étaient moins fréquents chez les diabétiques et presque un quart de nos diabétiques tuberculeux avait même une auscultation pulmonaire normale. La même tendance a été constatée également par d´autres auteurs [26]. Cette relative pauvreté clinique de la TPB+ chez les diabétiques par rapport aux non diabétiques pourrait découler de l´atténuation des réponses immunitaires au niveau pulmonaire chez les diabétiques comme rapportée par Wang et Sun [24,25]. Ainsi, nous préconisons aux soignants de penser à la tuberculose et de demander les explorations dédiées devant un désordre glycémique inexpliqué ou difficilement améliorable chez les diabétiques, même asymptomatiques avec un examen normal, dans un pays d´endémicité tuberculeuse comme le nôtre. Sur le plan biologique, la glycémie moyenne de nos TPB+ diabétiques avoisinait celle retrouvée au Pérou (259,55 mg/dl) [27]. Plus de la moitié d´entre eux avait un diabète très déséquilibré avec un taux d´HbA1c supérieur à 9%. D´un côté, l´hyperglycémie peut être due au déséquilibre du diabète induite par la tuberculose. Il s´agirait d´une ‘‘hyperglycémie de stress” faisant partie intégrante de la réponse inflammatoire. Elle est la conséquence d´un excès d´hormones de contrerégulation et de médiateurs inflammatoires entraînant une surproduction hépatique de glucose contrastant avec une diminution de son utilisation au niveau des tissus insulinodépendants [28]. De l´autre, les perturbations immunitaires liées à l´hyperglycémie persistante favoriseraient la survenue de la tuberculose. Ce qui constitue un cercle vicieux à rompre obligatoirement. Par ailleurs, ces diabétiques présentaient un syndrome inflammatoire biologique plus marqué par rapport aux non diabétiques puisque le diabète en soi s´accompagne déjà d´un état inflammatoire chronique [29] lequel s´amplifierait par l´infection. Leur fonction rénale était également moins bonne par rapport à celle des non diabétiques. Ceci s´expliquerait en partie par le fait que le diabète se complique fréquemment de microangiopathies dont la néphropathie diabétique [30,31]. L´adaptation posologique éventuelle du traitement antituberculeux doit tenir compte de cette fonction rénale. Une atteinte tuberculeuse rénale n´est pas à écarter également et qui pourrait participer à l´altération de la fonction rénale.

A la radiographie, les cavernes étaient, chez nous comme en Europe moins fréquentes chez les diabétiques par rapport aux non diabétiques. Ceci serait dû à l´altération de l´immunité à médiation cellulaire chez les diabétiques, laquelle aurait pour conséquence une perturbation des mécanismes de formation des cavernes, comme au cours de la coinfection tuberculose/Virus de l´Immunodépression Acquise (VIH) [32]. Par contre, les opacités systématisées qui sont des images plus en faveur d´une pneumopathie aiguë communautaire étaient beaucoup plus fréquentes chez les diabétiques de notre étude [33]. Ceci laisserait à penser que la tuberculose pulmonaire de nos diabétiques pourrait être associée à une surinfection bactérienne. Selon la littérature, le siège de prédilection de la tuberculose pulmonaire est le segment postérieur d´un des lobes supérieurs ou le segment apical du lobe inférieur puisque la pression partielle en oxygène y est la plus élevée, favorable au développement du BK [34]. La grande fréquence des lésions basales et des lésions étendues chez les diabétiques comparativement aux non diabétiques de notre étude était également constatée ailleurs comme en Chine [35], en Inde [36] et au Qatar [10]. Ceci pourrait être dû à l´immunodépression occasionnée par le diabète facilitant ainsi l´extension de la maladie.

**Limites de l´étude:** force est de reconnaître que notre étude présente des limites. En effet, elle n´a concerné que les patients présentant une TPB+ alors que des cas de tuberculose pulmonaire à bacilloscopie négative (TPB-) ont été enregistrés durant la période d´étude. De même, nous n´avons inclus que les patients hospitalisés. L´exclusion des cas de TPB- ainsi que de ceux traités en ambulatoire pourrait donc influencer nos résultats sur les principales manifestations clinico-biologiques et radiologiques de la tuberculose pulmonaire chez les diabétiques et les non diabétiques.

## Conclusion

La présente étude avait permis de constater que bien que les principales manifestations clinico-paracliniques soient typiques chez la plupart des diabétiques et non diabétiques hospitalisés pour TPB+, les diabétiques étaient en général plus âgés et avaient plus fréquemment un antécédent de tuberculose. Ils se faisaient alors hospitaliser plus tôt. Cliniquement, les asymptomatiques sur le plan respiratoire et ceux ayant une auscultation pulmonaire sans anomalie étaient plus fréquents tandis que ceux se plaignant d´anorexie et d´amaigrissement étaient moins fréquents. Biologiquement, leur syndrome inflammatoire était plus marqué. Radiologiquement, les opacités systématisées et des lésions de topographie basale ou étendue étaient plus fréquentes chez eux par rapport aux non diabétiques. Dans l´avenir, une étude sur les particularités évolutives de la TPB+ chez les diabétiques mérite d´être réalisée afin d´améliorer le pronostic de ces deux maladies.

### Etat des connaissances sur le sujet

- Le diabète augmente le risque de survenue de tuberculose par un facteur de deux à trois;
- Les présentations cliniques, biologiques et radiologiques de la tuberculose pulmonaire peuvent être plus sévères chez les patients diabétiques par rapport aux non diabétiques;
- Chez les diabétiques, il existe un risque accru de résultats défavorables au traitement antituberculeux.

### Contribution de notre étude à la connaissance

- Dans une population à faible revenu comme Madagascar, l´association entre la tuberculose et le diabète se révèle être plus fréquente et donc plus menaçante vues les limites techniques et économiques pour y faire face;
- L´âge supérieur à 45 ans, l´évolution clinique asymptomatique, le syndrome inflammatoire biologique important, les images radiographiques atypiques sont plus fréquents chez les diabétiques comparés aux non diabétiques.
